# Minimally invasive sacroiliac fusion: current insights and a comprehensive literature review

**DOI:** 10.55730/1300-0144.5898

**Published:** 2024-07-01

**Authors:** Aydın Sinan APAYDIN, Mehmet Denizhan YURTLUK, Khoi D. THAN

**Affiliations:** 1 Department of Neurosurgery, Faculty of Medicine, Karabük University, Karabük, Turkiye; 2 Faculty of Medicine, Bezmiâlem University, İstanbul, Turkiye; 3 Department of Neurosurgery, Faculty of Medicine, Duke University, Durham, NC, USA

**Keywords:** Sacroiliac joint, minimally invasive surgery, open surgery

## Abstract

**Background/aim:**

The sacroiliac joint (SIJ) is a frequently overlooked source of lower back pain (LBP). Recently, it has gained recognition as a significant pain generator, prompting increased interest in surgeries targeting this area. Traditionally, open SIJ fusion was used to stabilize pelvic and sacral fractures, and then it was adapted for use in pain management until the development of minimally invasive surgery (MIS). Revolutionizing the field, MIS offers fast and effective pain relief with significantly less risk of postsurgical adverse events, thereby gaining increased attention among surgeons. This review aims to highlight the current advancements in the literature regarding MIS SIJ fusion.

**Conclusion:**

The current literature demonstrates the superiority of MIS over open surgery with satisfactory patient outcomes and significantly lower complication rates. With the advancement in navigation and the inclusion of robotic assistance, MIS SIJ fusion is expected to become more ergonomically feasible for surgeons and shorten the learning curve for new trainees worldwide.

## 1. Introduction

The treatment of lower back pain (LBP) is a tremendous burden to healthcare systems worldwide [1]. The etiology and differential diagnosis of LBP may vary from myofascial disorders to spinal pathologies, including degenerative disc diseases, facet arthropathy, and spinal deformity [1,2]. In recent years, the sacroiliac joint (SIJ) has gained attention as a cause of LBP. It is estimated that the origin of 15%–30% of LBP is from SIJ pathologies, and research towards the management of these conditions has accelerated. LBP caused by SIJ pathology can be idiopathic in nature or stem from tumors, infections, degenerative diseases, rheumatologic diseases, pregnancy, trauma, extreme exercise, and prior spinal long-construct surgeries [1–8]. Since there are no attributed pathognomonic symptoms or history, it is paramount to do a thorough clinical history investigation, physical examination, and appropriate radiographical imaging to make a definitive diagnosis [9]. An ongoing debate in the literature has led to the proposal of combining several clinical history and physical examination findings that together might suggest an SIJ pathology. Patients typically present with pain in the buttocks that may radiate to the inguinal and flank regions as well as the upper legs. Also, the pain can be reproducible through several maneuvers including the flexion, abduction, and external rotation (FABER) test, distraction test, compression test, and Gaenslen maneuver [3,9,10,11]. It has also been suggested that a local anesthetic injection at the SIJ, leading to pain termination, can be used both as a diagnostic test and as a short-term medical treatment [3,12]. Patients who fail conservative management (e.g., local anesthetic injections and other medical strategies) and have pain for more than six months can be good candidates for surgical treatment with either open or minimally invasive surgery (MIS) to restore functionality and relieve pain [3,13,14]. Although open surgical fusion has been in our arsenal for decades, it is associated with significant surgical morbidity, high complication rates, and variable patient-reported outcomes; therefore, MIS methods have been gaining favor among surgeons, since they offer less soft tissue injury, reduced hospitalization time, comparable satisfactory fusion rates, and faster recovery times when compared with the open technique [15–22].

This review was conducted using specific keywords, including ‘sacroiliac fusion’, ‘minimally invasive surgery’, ‘sacroiliac joint’, and ‘robotic-assisted’. The search queries were applied to the databases PubMed, ScienceDirect, Scopus, and Web of Science. This review aims to explore the diagnosis, clinical decision making, and different treatment strategies in the management of SIJ pathologies in the current literature, focusing heavily on MIS.

## 2. Making the right diagnosis

The SIJ serves as a transfer point for the distribution of body weight between the spinal column and the hips [23]. This unique function can put significant mechanical stress on the joint and cause degenerative changes and dysfunction. Also, a significant portion of patients presenting with pain have a history of trauma from falls or motor vehicle accidents, prior lumbar surgery, pregnancy, or pain elicited by changing position or sitting [10,24]. Pain caused by the lumbar spine and SIJ can easily overlap and alter the direction of the diagnosis. To prevent this, several physical examination maneuvers combined with pelvic and lumbar computerized tomography (CT) or magnetic resonance imaging (MRI) studies can exclude alternative diagnoses, including nerve root compression, spondylolisthesis, infections, fractures, and malignancy, thereby pointing toward the SIJ as the source of the pain [25]. During physical examination, the FABER, distraction, compression, Gaenslen, and Mekhail tests can be used only to a certain degree to elicit pain and differentiate the SIJ as the source of pain from other causes since these tests don’t have the same sensitivity and generally have low specificity [3,9,10,11,26]. Unfortunately, none of these diagnostic measures have a high predictive capacity and are usually inconclusive. Therefore, diagnostic sacroiliac injections under fluoroscopic guidance with glucocorticoids or local anesthetic agents are starting to be used for both diagnosis and short-term pain relief [25,27,28]. In patients with high clinical likelihood, early use of SIJ pain-blocking injections is suggested to prevent unnecessary testing and provide quicker pain relief. When positive test results indicate the source of the pain is SIJ, fusion is generally the next clinical step [10,25]. It is vital to mention that diagnostic SIJ blocks have a high rate of false positive results, owing to extraarticular pain sources in the same region [29]. Due to the complex muscular and neurovascular anatomy of the lumbar and sacroiliac regions, it is paramount to do a thorough investigation before making a clinical diagnosis.

## 3. Open sacroiliac fusion

Open sacroiliac fusion was first described by Smith-Petersen [30] in the 1920s. It involves a curvilinear incision over the posterior two-thirds of the iliac crest followed by blunt dissection of the gluteus maximus muscle parallel to its fibers to create a flap and expose the lateral surface of the ilium. After sufficient exposure, a rectangular piece of bone is removed from the ilium to reveal the SIJ surface. Then, articular cartilage is curetted, and the bone piece is inserted into the joint space, creating an environment for arthrodesis and fusion. The fusion is stabilized using either T- or L-plate screws and the incision is closed properly. Patients are restricted from bearing weight on the affected side for the next three months or until a solid fusion is seen [16]. The open surgical approach provides improved functional outcomes and pain relief, but it also comes with certain disadvantages when compared to MIS techniques, including increased postoperative morbidity, longer recovery times, and reduced patient satisfaction [15,18–21]. Ballatori et al. [15], in their comparative analysis of open versus MIS approaches for SIJ fusion, found that patients who received open fusion for sacral pain had higher rates of novel postoperative pain and novel lumbar pathology within 30 days of follow-up. They also found that patients who received open fusion had higher rates of postoperative pain within 30 days of follow-up when compared to patients who had the MIS technique for sacroiliitis. For spondylosis, their study also revealed higher nonelective readmission within 30 days in the open technique group when compared to the MIS technique group. Also, the open technique resulted in higher rates of readmission due to infection in spondylosis patients and higher urinary tract infections in patients with sacral instability when compared to the MIS technique. Schütz et al. [18] reported poor outcomes following bilateral fusion with the open technique, with only 18% of their patients with SIJ diseases satisfied with the surgery. Smith et al. [21], in their multicenter comparative study, reported shorter operative and postoperative hospitalization times in the MIS group. Also, pain relief, calculated using visual analog scale (VAS) score changes from the baseline to 12 months postoperative, was greater in the MIS group compared to the open surgical group. When the groups were matched for age, gender, and history of prior lumbar surgery, the MIS group had lower postoperative pain scores compared to the open surgical group.

## 4. Minimally invasive surgery

MIS fusion emerged in 2008 as a novel technique to reduce operative time, blood loss, morbidity, and associated complications, leveraging intraoperative navigation [9,31]. Since then, several techniques and implant types have been developed to increase fusion rates and patient satisfaction. The lateral transiliac and posterior techniques were the first to develop; subsequently, several other novel variations of the previous two techniques have been formulated. Additionally, pin placement guidance has evolved from the use of fluoroscopy to obtain anterior, lateral, pelvic inlet, pelvic inlet-oblique, pelvic outlet, and pelvic outlet pubic views to the use of CT-guided navigation with reference frames. This latter approach has gained widespread adoption among surgeons.

### 4.1. Lateral technique

The lateral technique has been the most utilized technique for sacroiliac fusion since the introduction of MIS by Wise and Dall [31]. In the lateral technique, the ilium is accessed by a small incision under fluoroscopic guidance, and screws are placed from the lateral to the medial, from the ilium to the sacrum medially crossing the SIJ for fixation, generally using two or three screws in the procedure [32]. Several authors described the procedure in detail, and despite minor differences, the basic principles remained the same [33,34]. The authors emphasized that obtaining a preoperative pelvic CT scan is crucial to planning an appropriate approach to the SIJ. On the operative day, patients are first placed in a prone position under general anesthesia. Generally, lateral, pelvic inlet, and pelvic outlet views are obtained using fluoroscopy to determine an appropriate starting point. Pelvic inlet and outlet views can be determined using the preoperative pelvic CT scan and the lateral sacral view for guidance. Identifying the true pelvic inlet and outlet views is of utmost importance for SIJ fusion. Krappinger et al. [35] described a free-hand technique in their study showing how to find the true pelvic inlet and outlet views using preoperative pelvic CT scans in a step-by-step fashion. Firstly, the midsagittal plane is applied on the sagittal window. Then, the coronal axis is adjusted parallel to the posterior border of the S1 vertebrae and the horizontal plane is adjusted parallel to the caudal border of S1. Next, the window is switched to inlet view and the sagittal axis is again adjusted for the true midsagittal plane. To understand the correct placement, two anatomical landmarks can be found in the image: the symphysis pubis and the spinous process. In a true pelvic inlet view, the midsagittal plane must cross the symphysis pubis anteriorly and the spinous process posteriorly. For the pelvic outlet view, the outlet window is opened, and the horizontal axis must be parallel to the cranial borders of the SIJ and cross at the centers of the S1, and the vertical axis of the sacrum must be parallel to the midsagittal axis [35]. These steps provide the guidance and the means to ensure a solid fusion and decrease complication rates. After obtaining adequate imaging, an incision is made lateral to the sacral midline, and a pin is positioned in the correct location using the lateral view and guided through the ilium and SIJ using the guidance of the inlet and outlet views. Following this, an implant of the right size is chosen and a drill is passed through. The shape of the broach is dependent on the shape of the implant. There are several implant options in the market, such as triangular titanium (iFuse, SI-BONE, Inc., Santa Clara, CA, USA), cylindrical (Rialto, Medtronic, Minneapolis, MN, USA), and bone grafts with threaded implants (SImmetry Sacroiliac Joint Fusion System, Zyga Technology, Minnetonka, MN, USA) [36–38]. In the lateral technique, the sacral ala is the initial location for the first implant and subsequent implants inserted caudally, the second implant being adjacent or above the S1 foramen and the third between the S1 and S2 foramen [33]. The number of implants varies depending on the patient’s anatomy. As the last step, the surgery site is irrigated, and the incision is closed in a layered fashion. Despite its benefits, the lateral technique does have some drawbacks. The lateral technique requires dissection of the gluteal fascia and muscles, which carries the risk of injuring the cluneal nerves and the superior gluteal artery [39]. Also, there are reported cases of S1 and S2 nerve root compression and irritation presenting with novel postoperative pain associated with the lateral to medial trajectory of the implants [39,40]. Therefore, choosing the right depth and size for the implant is of crucial importance.

### 4.2. Posterior technique

As the name suggests, the posterior technique approaches the SIJ more posteriorly and achieves fusion by implanting the implants in an oblique trajectory. The posterior technique was first developed to alleviate the risk of neurovascular injury and nerve root irritation, including the S1 and S2 nerves and the superior gluteal artery, most prominently seen in lateral fusion surgeries [39]. However, the posterior technique has the disadvantage of dissecting through the SIJ ligaments, and the hardware is implanted in a more longitudinal trajectory [40]. Raikar et al. [40] described the posterior oblique trajectory, which provides a more direct fusion of the SIJ without dissecting the gluteal soft tissue and SIJ ligaments through the ileum and offers less risk of neurovascular and soft tissue injury. In further detail, in the posterior oblique technique, a pin is inserted through the upper outer surface of the iliac crest at the level of the superior surface of the sacral ala using a mallet under fluoroscopic guidance. Following confirmation of appropriate needle placement, a guide wire is inserted followed by a longitudinal incision around the guide wire. A cannula is inserted with the help of a tissue dilator and a screw is inserted through SIJ, aiming for the anterior superior surface of the sacral ala. Subsequent implants follow the same principles and are inserted 1.5 cm caudally in a parallel trajectory, and the incision is closed in a layered fashion.

A posterior interposition or intraarticular technique is also described where fusion is achieved through ligamentotaxis for early or short-term joint stabilization or for distraction arthrodesis in the case of long-term SIJ fusion [39]. However, there is limited evidence in the current literature. Similarly, Lynch et al. [41] described a similar approach called ‘inferior, intraarticular arthrodesis’, where the SIJ is approached at the level of or above the posterior superior iliac spine (PSIS) or at a trajectory inferior to it. Unlike the lateral or other posterior techniques, which use metallic implants, the intraarticular approach uses bone allografts to promote bone stabilization. The bone allograft covers a greater surface area than metallic implants within the SIJ, thereby providing a greater chance of fusion [41]. In the superior intraarticular trajectory, the bone allograft is implanted in the ligamentous portion of the SIJ parallel to the S1 end plate, while in the inferior intraarticular trajectory, the bone allograft is only in the articular portion of the SIJ, providing a better chance of stabilization [41]. Also, having the bone allograft placed adjacent to the sacral axis of rotation may induce more robust stabilization and minimize the biomechanical forces applied to the implant in daily motion [41]. This technique offers promising results, even though it still lacks real-world clinical and long-term follow-up data.

## 5. Discussion

The SIJ is a common cause of low back pain, with a wide variety of differential diagnoses. It can easily overlap with other causes in symptomatology, therefore making it overseen as a cause and easier to miss as a diagnosis. Several diagnostic tests in the form of physical examination maneuvers and pain management with pharmacologic agents can help make the diagnosis easier and guide the physician in the right direction. Conservative therapy with antiinflammatory agents and short-term relief with pain blockers is the initial next step upon diagnosis, but these measures generally fail within 6 months, making SIJ fusion the next option.

Classically, SIJ fusion was first described by Smith-Petersen [30] in the 1920s to address pelvic and sacral fractures and stabilize the pelvic structure through open SIJ fusion. MIS SIJ fusion was developed in 2008 to address the shortcomings of open fusion. Since then, several techniques have been developed to improve patient-reported outcomes and decrease surgical complications. Several studies found significantly improved VAS and Oswestry disability index (ODI) scores at 6, 12, 18, and 24 months postoperatively compared to the baseline for MIS alone or when compared to open fusion [21,42]. Vanaclocha-Vanaclocha et al. [42] followed 24 patients who had undergone MIS SIJ fusion for multiple years and found that, using VAS scores as an indicator of pain relief, patients experienced the most benefit from the surgery at 18–24 months postoperatively. In the same study, they also prospectively assessed ODI scores and reported decreased ODI scores compared to baseline. Mao et al. [43] compared patients with prior lumbar fusion surgery and nonlumbar fusion surgery who underwent MIS SIJ fusion. They have found that patients with prior lumbar fusion surgery were more prone to report higher ODI scores postoperatively compared to patients with no prior lumbar fusion. Using metaanalysis, Mehkri et al. [9] analyzed the outcomes of 34 studies and found that both VAS and ODI scores improved at 6, 12, 18, and 24 months postoperatively compared to the baseline.

Mehkri et al. [9] also compared fusion rates, confirmed with CT and/or oblique radiography; among nine studies only one reported a fusion rate of <75% [44]. Fuchs et al. [44] associated this low fusion rate with inadequate curettage of the joint surface for the bone allograft, unfavorable positioning of the implant, and early assessment of the surgical results since the formation of bone bridges across the SIJ takes time.

Malpositioning of the implant is the most common reason for revision surgery following MIS SIJ fusion. The average revision rate was 3.2% across 24 studies [9]. Smith et al. [21] compared the revision rates between open and MIS surgeries and found a 43% revision rate in the open surgeries due to implant loosening, spinal nerve root implant irritation, and pseudoarthrosis; however, the revision rate in the MIS group was only 3.5%. Several other studies that compared open and MIS surgeries also reported higher revision rates in the open cohorts, showing the superiority of the MIS technique over open surgery [22,45,46]. Complications following MIS SIJ fusion tend to be minimal to none. Vanaclocha-Vanaclocha et al. [42] reported four patients with immediate postoperative pain that resolved with adequate antiinflammatory medications. Mehkri et al. [9] also found that complication rates were minimal and postoperative pain could be adequately addressed with antiinflammatory medications. Other studies reported postoperative pain due to implant malpositioning that did not require revision, fluid retention, trochanteric bursitis, and facet pain [42,47,48]. Some studies reported severe complications, including persistent lower back pain, nerve impingement, radiculitis, and neurologic deficits, due to screw mispositioning that required revision surgeries [44,49,50].

The future of MIS SIJ fusion lies in robot-assisted surgery with navigation. Lee et al. [51] compared robotic and nonrobotic sacroiliac fusion surgeries. They concluded that robotic surgery was associated with less radiation exposure due to the need for fewer fluoroscopic images during the surgery, thereby positively impacting surgical teams over their lifetimes while offering similar results in terms of fusion rates and patient satisfaction.

## 6. Conclusion

The SIJ has gained significant attention as one of the major causes of lower back pain. The history of SIJ fusion remained stable until the discovery of MIS. This new area offers patients a superior treatment option over the open technique with significantly lower complication rates and hospitalization times. This new area has been opened to innovation and advancement with the integration of robotic surgery techniques.
